# Drainless Robotic Resection of Mediastinal Lesions: A Case Series of Three Patients

**DOI:** 10.7759/cureus.108127

**Published:** 2026-05-02

**Authors:** Aysegul Kurtoglu, Mahir Fattahov, Ilknur Aytekin Celik, Muhammet Sayan, Ali Celik

**Affiliations:** 1 Department of Thoracic Surgery, Faculty of Medicine, Gazi University, Ankara, TUR; 2 Department of Thoracic Surgery, Faculty of Medicine, Yildirim Beyazit University, Ankara, TUR

**Keywords:** chest tube, drainless, mediastinal mass, robotic surgery, thoracic surgery, tubeless

## Abstract

Robotic surgery has become increasingly adopted in minimally invasive thoracic procedures because of its advantages, including three-dimensional intraoperative visualization, reduced tissue trauma, and reduced intraoperative bleeding. In line with Enhanced Recovery After Surgery (ERAS) principles, drainless surgical approaches have recently gained attention as they may help reduce postoperative pain and support earlier discharge. In this case series, we describe retrospective cases of three patients who underwent drainless robotic resection of mediastinal lesions at our institution. With careful patient selection, this approach may offer several potential benefits, including reduced postoperative pain, shorter hospital stay, and faster recovery. Nevertheless, close postoperative monitoring remains essential, and the potential need for thoracic drainage should always be kept in mind.

## Introduction

The first robot-assisted thoracic surgery (RATS) procedure was reported by Melfi et al. in 2002 [1]. Subsequent studies have demonstrated that RATS is associated with reduced intraoperative bleeding and allows less traumatic surgical manipulation compared with conventional approaches [2]. Chest tubes routinely placed after thoracic surgery have been shown to increase postoperative pain and negatively affect respiratory function and exercise capacity [3]. With the advancement of minimally invasive surgical techniques and the implementation of Enhanced Recovery After Surgery (ERAS) protocols, the routine use of postoperative thoracic drainage has increasingly been questioned. In this study, we present three cases of robotic mediastinal mass resection performed without postoperative chest tube placement at our institution.

## Case presentation

Three female patients who underwent drainless robotic resection of mediastinal lesions at our institution within the past year were retrospectively evaluated. The three patients were aged 39, 45, and 47 years, presenting with various clinical complaints.

Despite differences in medical history, all procedures were performed under elective conditions. Port placement was determined according to the anatomical localization of each lesion. After confirming complete lung expansion and the absence of air leakage, the procedures were completed without chest tube placement. No complications were observed during the early postoperative period in any of the patients.

Case 1

A 39-year-old female patient was admitted to our clinic with back pain. Her medical history was significant for gastroesophageal reflux disease. Imaging revealed a paravertebral cystic lesion, and the patient was followed with a preliminary diagnosis of a mediastinal cyst. A thoracic CT performed in 2024 demonstrated a lesion measuring 30 × 15 mm, and follow-up imaging showed an increase in size. The patient was referred to our clinic, and robotic surgical resection was performed with the preliminary diagnosis of a mediastinal cyst (Figure 1).

**Figure 1:**
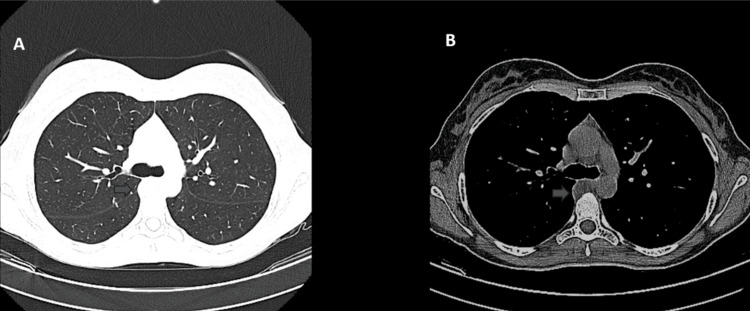
Computed tomography images of Case 1. The lesion is indicated by the arrow. (A) Thoracic CT, lung window, paravertebral mass (arrow). (B) Thoracic CT, mediastinal window, paravertebral mass (arrow).

Case 2

A 45-year-old female patient with myasthenia gravis (MG) was referred to us for an anterior mediastinal mass. Her medical history was significant for anxiety disorder. For further evaluation, positron emission tomography-computed tomography (PET-CT) was performed. PET-CT demonstrated a mass approximately 3 cm in size located in the anterior mediastinum in the left paramedian region, with a maximum standardized uptake value (SUVmax) of 3.4. Robotic surgical resection was performed with a preliminary diagnosis of thymoma. Histopathological examination revealed type AB thymoma (Figure 2).

**Figure 2:**
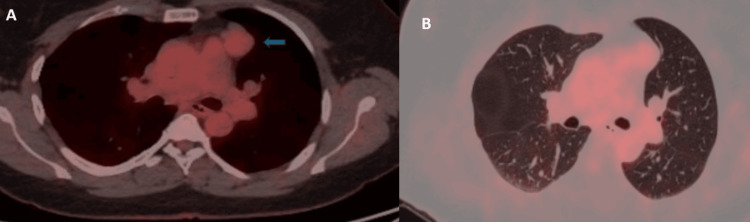
Positron emission tomography-computed tomography (PET-CT) images of Case 2. The lesion is indicated by the arrow. (A) Axial FDG PET-CT image (mediastinal window) showing an FDG-avid soft tissue lesion (arrowed) located in the left paramedian anterior mediastinum. (B) Axial FDG PET-CT image (lung window) showing the relationship of lesion with the surrounding lung parenchyma. Orientation: The right side of the image represents the right side of the patient, and the left side represents the left side (standard radiological view).

Case 3

A 47-year-old female patient with Budd-Chiari syndrome was admitted to the department of emergency surgery with dyspnea, palpitations, and chest pain complaints. Her medical history was significant for hypertension. Thorax CT showed a mediastinal mass at the paracardiac region. Cardiac magnetic resonance imaging (MRI) revealed a cystic lesion approximately 9 cm in size in the anterior mediastinum adjacent to the anterior wall of the left ventricle. The patient was referred to our clinic, and robotic surgical resection was planned with a preliminary diagnosis of thymic cyst. Histopathological examination confirmed the diagnosis of thymic cyst (Figure 3). Table 1 presents the summary of patient demographics, clinical characteristics, and surgical outcomes.

**Figure 3:**
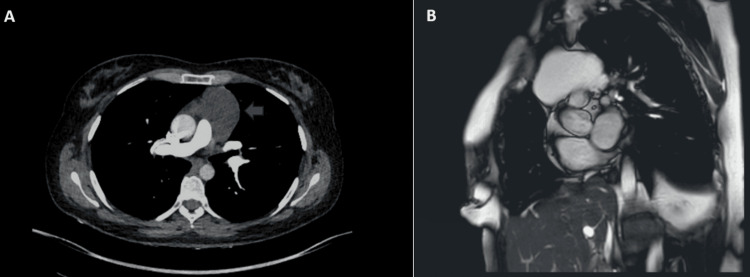
Computed tomography and magnetic resonance imaging of Case 3. The lesion is indicated by the arrow. (A) Thoracic CT, mediastinal window, demonstrating a pericardial cystic lesion (arrow). (B) Cardiac MRI: pericardial cystic lesion.

**Table 1:** Summary of patient demographics, clinical characteristics, and surgical outcomes. Note: For Case 2, an additional small utility incision was performed solely for specimen extraction; the surgical dissection was completed entirely with a three-arm robotic configuration. GERD: Gastroesophageal reflux disease; MG: Myasthenia gravis; RATS: Robotic-assisted thoracic surgery; Pnx: Pneumothorax; min: minutes; mm: millimeters

Feature	Case 1	Case 2	Case 3
Age/Gender	39/Female	45/Female	47/Female
Comorbidities	GERD	MG, Anxiety disorder	Budd-Chiari, Hypertension
Symptoms	Back pain	Referred (Asymptomatic)	Dyspnea, Palpitation, Chest pain
Lesion Location	Posterior Mediastinum (Paravertebral)	Anterior Mediastinum (Left paramedian)	Anterior Mediastinum (Paracardiac)
Imaging Size (mm)	30 x 15	~30 (3 cm)	~90 (9 cm)
Surgical Approach	Right-sided RATS (3-arm)	Left-sided RATS (3-arm*)	Left-sided RATS (3-arm)
Operative Time (min)	120	180	210
Histopathology	Mediastinal Cyst	Type AB Thymoma	Thymic Cyst
Postop. Complication	None	Minimal Pnx (Resolved)	None
Clavien-Dindo Grade	Grade 0	Grade I	Grade 0

Surgical technique

All patients were intubated with a 35 fr left-sided double-lumen endotracheal tube, and positioning was performed with elevation of the hemithorax containing the lesion. Before port placement, 0.25% bupivacaine was infiltrated into the planned incision sites for preemptive analgesia. Three 8-mm robotic working ports were used: (1) fifth intercostal space at the midclavicular line; (2) sixth intercostal space at the midaxillary line; and (3) third intercostal space at the anterior axillary line.

Intraoperative exploration confirmed that all lesions were extrapulmonary. The lesions were dissected from the surrounding tissues using Cadier forceps and a Maryland dissector (Figure 4). The procedures were performed using a three-arm robotic approach with CO_2_ insufflation at a pressure of 6-8 mmHg.

**Figure 4:**
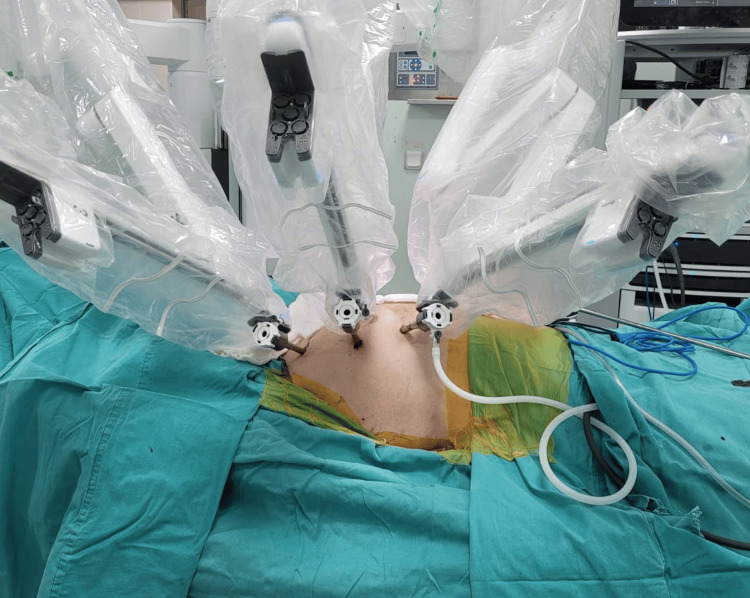
Intraoperative robotic view of the right-sided mediastinal lesion. Orientation: The left side of the image represents the cranial direction, and the right side represents the caudal direction.

In two patients, the specimen was removed by enlarging the working port at the midclavicular line. In the thymoma case, an additional subxiphoid utility incision was created to reduce postoperative pain, and the lesion was removed using an endobag (Figure 5).

**Figure 5:**
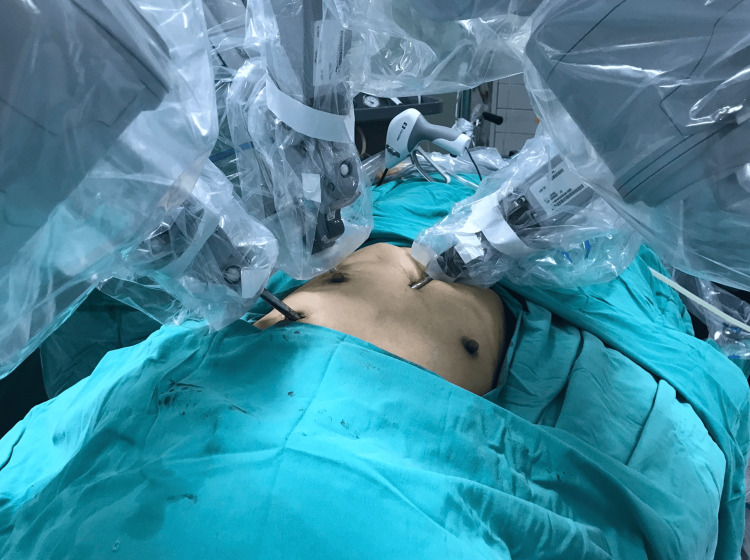
Intraoperative robotic view of the left hemithorax. The anatomical orientation is as follows: the bottom of the image represents the cranial (superior) direction, while the top represents the caudal (inferior) direction.

Complete excision was achieved in all cases. The operative times for Case 1, Case 2, and Case 3 were 120, 180, and 210 minutes, respectively. Intraoperative blood loss was minimal in all cases, and no patient required a blood transfusion. As no suspected parenchymal injury or intraoperative complications were observed, the procedures were planned to be completed without chest tube placement. A 16-Fr catheter was temporarily inserted through one of the working ports and connected to a controlled negative-pressure underwater drainage system. A negative pressure of -10 cmH₂O was applied until active air evacuation ceased. Simultaneously, the anesthesia team performed a recruitment maneuver to reopen collapsed alveoli and achieve full lung expansion. After confirming the absence of active air leakage via direct observation of the underwater seal system (observing no air bubbles during the recruitment maneuver) and no ventilatory leak during mechanical ventilation, the catheter was removed, and the incisions were closed (Figure 6).

**Figure 6:**
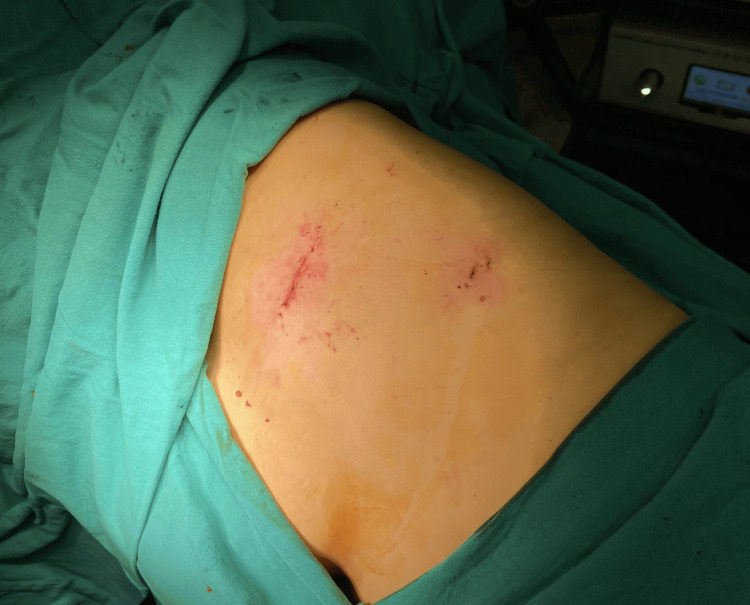
Lateral view of the thoracic wall of Case 3 postoperatively without a chest tube. Postoperative view of the left hemithorax in a patient who underwent robotic resection of a left mediastinal mass. Orientation: The patient is in the left lateral decubitus position. The left side of the image represents the cranial direction, and the right side represents the caudal direction.

Postoperative follow-up

After surgery, patients were closely monitored in the recovery unit with particular attention to vital parameters and the development of subcutaneous emphysema. Oral intake and mobilization were initiated at the sixth postoperative hour. Radiological evaluation was performed with a chest X-ray at the first postoperative hour. Postoperative complications occurring within 30 days of surgery were evaluated according to the Clavien-Dindo classification, which ranks surgical complications based on the therapy required for their management [4]. In this grading system, Grade I complications are defined as any deviation from the normal postoperative course that does not require pharmacological, surgical, endoscopic, or radiological intervention [4].

In our series, two patients had an uneventful recovery (Grade 0). In one patient, a minimal apical pneumothorax was detected. A follow-up chest X-ray obtained at the sixth postoperative hour confirmed that there was no progression of the pneumothorax. Following the Clavien-Dindo criteria, this patient was classified as Grade I, as the condition was managed conservatively with supplemental oxygen and daily monitoring; the pneumothorax resolved spontaneously by the first postoperative day (Figure 7) without the need for chest tube insertion. Patients were discharged between postoperative days one and three. During follow-up, no complications or hospital readmissions were observed.

**Figure 7:**
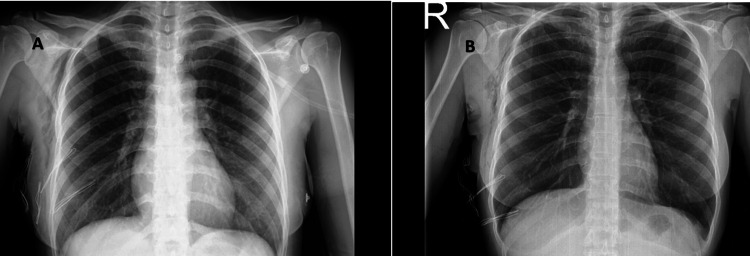
Postoperative chest X-rays. (A) Chest X-ray obtained at one hour postoperatively, showing a minimal apical pneumothorax and mild subcutaneous emphysema. (B) Chest X-ray from postoperative day one, demonstrating significant resolution of both the pneumothorax and the subcutaneous emphysema without the need for additional intervention.

## Discussion

Chest tube placement following thoracic surgery has been routinely performed since the 1950s. With the evolution of surgical techniques, the initial practice of double chest tube placement was gradually replaced by single chest tube drainage. More recently, with the implementation of ERAS protocols, we successfully managed three robotic mediastinal resection cases without routine postoperative thoracic catheterization [5,6].

Although several studies have investigated drainless lung resections, data regarding drainless mediastinal mass excision remain limited. Previous reports have shown that drainless robotic thymectomy and esophagectomy can be safely performed without significant postoperative complications, such as pneumothorax or pleural effusion. These studies also reported reduced postoperative pain, shorter hospital stay, and faster recovery in patients undergoing drainless procedures [7]. In line with these findings, recent studies on drainless video-assisted thoracoscopic surgery (VATS) have further demonstrated that such approaches can significantly reduce hospital stays by up to 30% and healthcare costs [8].

Robot-assisted surgery provides high-resolution three-dimensional visualization, tremor filtration, and enhanced instrument articulation, enabling more precise and controlled tissue dissection. These technical advantages reduce mechanical trauma to surrounding tissues and may help minimize intraoperative bleeding and parenchymal injury. Considering the stability and precision provided by robotic surgery, this technique may offer a technically favorable environment for drainless surgical approaches. In our case series, the successful completion of robotic mediastinal mass excisions without chest tube placement supports this hypothesis.

Our team routinely performs robotic lung resections and mediastinal surgeries. We have extensive experience with drainless VATS, which we have successfully transitioned into our robotic practice. We also have experience in drainless robotic lung resections. In this series, we compiled our cases of drainless robotic mediastinal mass excision. Success in this approach depends on the surgeon's technical proficiency in robotic surgery, mastery of drainless surgical procedures, appropriate postoperative care, and the ability to manage potential complications effectively. In this context, careful postoperative monitoring remains essential to ensure patient safety and to allow for timely intervention if any clinical issues arise.

## Conclusions

This case series represents one of the limited reports describing drainless robotic mediastinal lesion excision. A drainless approach to minimally invasive mediastinal surgery is considered a safe and feasible technique when implemented with careful intraoperative assessment. This strategy, often employed in VATS or RATS, generally results in decreased postoperative pain, faster recovery, and high patient satisfaction. The successful outcomes in our series suggest that this approach is a potentially safe and feasible option in robotic mediastinal surgery. However, due to the limited number of patients, larger studies are required to further validate these findings and define the optimal patient population for this approach.
